# Causality‐Preserving Generative Adversarial Networks for the Diagnosis of Neurological Disorders

**DOI:** 10.1049/syb2.70085

**Published:** 2026-09-30

**Authors:** Junhong Ren, Wensheng Zhang

**Affiliations:** ^1^ School of Computer Science and Engineering Nanjing University of Science and Technology Nanjing China; ^2^ Institute of Automation Chinese Academy of Sciences Beijing China

**Keywords:** biomedical MRI, brain models, causality, neural nets

## Abstract

The brain network has proven to be an effective tool for recognising neurological disorders. However, current brain network modelling algorithms often rely on numerous assumptions when evaluating the interaction relationships between brain regions, which are frequently shown to be inaccurate. For instance, some studies assume that interactions among brain regions are linear. Additionally, some research posits that other brain regions do not influence the evaluation outcomes when assessing causal effects among paired brain regions. To address this challenge, we propose an innovative method for modelling brain networks that estimates interaction relationships among brain regions from a causal perspective. Notably, during the evaluation process, this method comprehensively considers all relevant brain regions rather than examining individual relationships in isolation. To validate the efficacy of our approach, we conducted extensive experimental verifications using publicly available datasets. The experimental results indicate that our proposed brain network modelling method not only demonstrates superior performance in the dementia identification task but also successfully identifies several new biomarkers, providing novel perspectives and insights for dementia research.

## Graphical abstract

Existing brain network modelling algorithms for identifying neurological disorders suffer from inaccurate assumptions, such as linear inter‐brain‐region interactions and ignoring the influence of other regions when evaluating paired causal effects. To address this, we propose an innovative causal‐perspective method that comprehensively considers all relevant brain regions, and extensive experiments on public datasets confirm its superior performance in disorder identification and ability to discover new biomarkers, offering new insights for neurological disorder research.

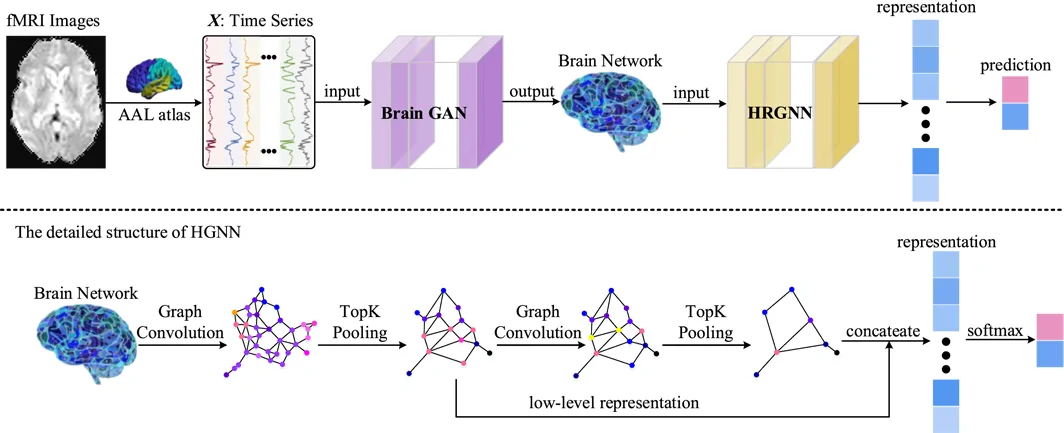

## 1 Introduction

Alzheimer's disease (AD), a prevalent neurological disorder, affects tens of millions of individuals globally and has emerged as a significant public health concern [1, 2]. Despite extensive research, scientists have yet to develop an effective clinical treatment for this disease. Mild cognitive impairment (MCI) is the transitional phase between the cognitive decline associated with normal ageing and the more severe cognitive decline observed in dementia. A large number of studies indicate that patients with MCI face a significantly increased risk of developing AD, with more than half showing a high likelihood of progressing to AD within 5 years [3, 4]. Various clinical studies have demonstrated that early interventions for MCI patients, such as pharmacotherapy and cognitive training, can delay the progression of their condition to some degree, thereby reducing their risk of ultimately developing AD [5]. Consequently, the accurate diagnosis of MCI has attracted considerable attention and has become a prominent topic of academic research in recent decades. However, identifying MCI remains challenging due to the subtle differences between MCI patients and individuals experiencing normal ageing [6].

Functional magnetic resonance imaging (fMRI) technology, as a non‐invasive tool, has demonstrated significant potential for diagnosing neuropsychiatric disorders, providing robust support for in‐depth analysis of brain activity [7]. Numerous research findings indicate that functional brain networks can effectively capture the temporal correlation characteristics between different brain regions, thereby identifying regions closely associated with functional impairments caused by neurological disorders [8, 9]. In the field of brain imaging analysis, various brain network modelling strategies have been proposed, which can be classified into three categories: Pearson's correlation (PC) brain network [10], high‐order brain network [11], and sparsity brain network [12]. Specifically, PC‐based methods, which are a mainstream approach for estimating functional brain networks, quantify the interaction strength between brain regions by calculating the correlation of blood oxygenation level‐dependent (BOLD) signals between pairs of regions. However, brain networks constructed using PC often exhibit high density, making it challenging to precisely identify connections most relevant to diseases [13]. As an extension of PC‐based methods, higher‐order brain networks also tend to generate dense graph structures. In contrast, sparse brain network methods can more distinctly display strongly correlated interaction patterns by eliminating weakly correlated connections. Several studies demonstrate that sparse brain networks are more effective for identifying brain disorders [14, 15].

However, it is important to note that the methods mentioned above are limited to using correlation to define the interactive relationships between brain regions. A fundamental limitation of these approaches is their inability to effectively determine the specific directionality of signal transmission between brain regions. As a highly complex neural system, the internal transfer of information within the brain relies heavily on neuronal activity and connectivity patterns [16]. This indicates that a brain network modelling framework that incorporates directional factors is essential for accurately representing the interaction patterns between brain regions. Given the critical role of signal transmission directionality in understanding brain organisation rules, causal inference methods are increasingly valued by researchers in the field of brain network modelling, as they allow for a clear distinction of signal flow paths. Advances in this field have led to the development and implementation of various causal brain network modelling algorithms, with Granger causality (GC) being one of the most widely used methods; it assumes that upstream signals are replicated by downstream signals. Time lag is often considered, but this assumption is frequently invalid in fMRI due to acquisition noise [17]. Bayesian networks (BN), used to construct brain effective connectivity networks (ECNs), require candidate graphs to be directed acyclic graphs (DAGs), yet brain functional interactions are not acyclic due to reciprocal polysynaptic connections [18, 19]. The data‐driven linear non‐Gaussian acyclic model (LiNGAM) infers information flow via independent component analysis (ICA), but ICA needs large time series data, leading to poor LiNGAM performance with small fMRI samples [20].

To address the limitations of current approaches to dementia recognition, we propose a novel framework that employs a Generative Adversarial Network (GAN) to simultaneously estimate the causal effects among various brain regions, as illustrated in Figure 1. Furthermore, to improve recognition performance for brain disorders, we have implemented a popular graph pooling layer to generate hierarchical representations of brain networks. Experiments conducted on the Alzheimer's Disease Neuroimaging Initiative (ADNI) database demonstrate the superiority of our proposed method compared with existing techniques. In summary, the proposed framework offers several key advantages:

- The proposed GAN is a flexible approach for inferring causal effects between brain regions using an end‐to‐end framework.
- The employed graph pooling layer can capture critical substructures of the brain network, enhancing performance in the identification of dementia.
- The estimated brain network generated by our method demonstrates nonlinear interactions between brain regions, surpassing traditional linear approaches.

**FIGURE 1:**
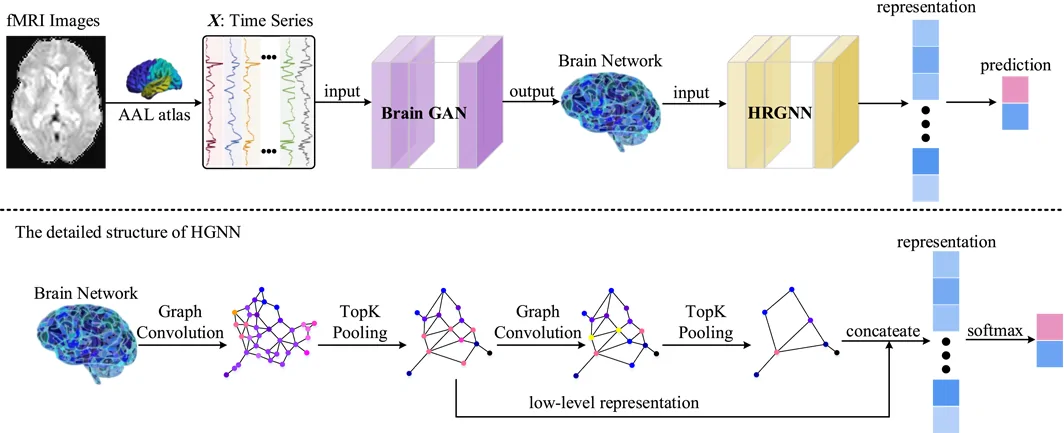
Illustration of the proposed framework for dementia identification. Initially, signals from each brain region are extracted using the Automated Anatomical Labelling (AAL) atlas. Subsequently, the extracted time series data, referred to as X, is then input into the Brain GAN model to estimate the causal effects among brain regions, resulting in the generation of a brain network as the output. Ultimately, the estimated brain network is fed into the HGNN to obtain the prediction results.

The structure of this paper is organised as follows: First, in Section 2, we review the most relevant techniques related to this study. Next, we provide details of the brain network estimation method and the updating rules for the graph pooling layer in Section 3. Following this, the materials and procedures involved in model training are presented in Section 4. In Sections 5 and 6, we present the experimental results and findings of this study. Finally, we summarise the research outcomes in Section 7.

## 2 Related Work

In this section, we first summarise the recent advances in brain network estimation in Section 2.1, and then review the progress of graph neural networks (GNNs) in the recognition of neurological disorders in Section 2.2.

### 2.1 Causality for Brain Network Estimation

Granger causality and its variants are effective algorithms for determining causal relationships between variables [21]. The fundamental hypothesis is that if the past values of a time series X can significantly improve the predictive accuracy of future values of another time series Y, then X is considered to Granger‐cause Y. Over the past several decades, numerous researchers have utilised Granger causality and its variants to investigate interaction patterns between brain regions, yielding many significant discoveries. For example, Su et al. developed an advanced nonlinear Granger causality algorithm for constructing brain networks [22]. Their study revealed that patients with amnesic mild cognitive impairment exhibited a significant reduction in both global efficiency and the average clustering coefficient of their brain networks compared with healthy individuals. Yi et al. employed a novel algorithm called nonparametric Granger causality to evaluate time‐varying causal effects among brain regions [23]. They discovered significant differences in brain activity patterns between individuals with left hemiplegia and those with right hemiplegia.

Despite the numerous achievements of Granger's algorithm in the field of brain network modelling, it still exhibits two notable limitations. First, these algorithms can only determine the presence of a causal relationship between brain regions in a binary format (0 and 1), lacking the capability to provide a more precise quantitative assessment of the strength of causal effects. Second, they typically employ a pairwise matching approach to estimate causality, often neglecting the potential influence of other brain regions on the relationship between the two regions being analysed. These limitations significantly contribute to the inaccuracy of the causal effects between brain regions. To address the aforementioned issues, we have developed a novel generative adversarial network (GAN) designed to estimate the causal effects between brain regions. This network integrates all relevant elements for simultaneous analysis.

### 2.2 Graph Neural Network for Brain Disorder Identification

Brain networks illustrate the interaction patterns among various brain regions as graphs, creating a highly interconnected structure. Due to the significant advantages of graph neural networks (GNNs) in addressing reasoning tasks that involve graph‐structured data, numerous GNN models have been developed and applied in the field of neurological disorder identification. Specifically, Zhang et al. developed a novel GNN to improve the performance of traditional recognition frameworks by integrating additional clinical information [24]. The proposed model achieved optimal classification performance on both public datasets. Zheng et al. proposed an interpretable GNN model for the recognition of neurological disorders, which exhibited outstanding performance on both simulated and real datasets [25].

Numerous studies have demonstrated that neurological disorders primarily result in localised abnormalities in brain function, rather than affecting all brain regions comprehensively [11, 12, 15]. However, current classification frameworks based on GNNs overlook the significance of these localised impacts in their design. Consequently, it is essential to accurately identify the substructures of brain networks that are most closely associated with neurological disorders. To address this limitation, we have integrated graph pooling operations into the proposed framework to extract hierarchical representations of brain networks, thereby improving the recognition performance of downstream tasks.

## 3 Methodology

In this section, we first present the problem formulation in Section 3.1. Next, the methodology for brain network estimation is introduced in Section 3.2. Finally, the updating rule for hierarchical graph representation learning is explained in Section 3.3.

### 3.1 Problem Formulation

The identification of dementia patients can be conceptualised as a graph classification task, aimed at training a model to detect cognitive function abnormalities in patients with brain disorders. Let $G_{\Delta }=\left\{G_{1},G_{2},\text{…},G_{n}\right\}$ represent a set of brain networks, where $G_{i}$ denotes the brain network of the i‐th subject. Let $Y\in R^{n\times c}$ represent the corresponding labels for each brain network. If $G_{i}$ belongs to the j‐th class, then $Y_{i,j}=1$; otherwise, $Y_{i,j}=0$. The training set for the graph classification task can be formulated as follows:

(1) $$D=\left\{\left(G_{i},Y_{i}\right)|G_{i}\in G_{\Delta },Y_{i}\in Y,1\leq i\leq L_{D}\right\}$$

where $G_{i}$ is an input sample and $Y_{i}$ denotes the corresponding ground truth label. $L_{D}$ is the number of samples in the training set. The primary objective of the graph classification task is to obtain a function $F\left(G_{i}\right)\to Y_{i}$ from D that accurately maps each sample to its respective label. The objective is to minimise the cross‐entropy between the ground truth labels and the output, which can be expressed as follows:

(2) $$Loss=-\sum_{i\in L_{D}}\sum_{j=1}^{c}Y_{i,j}\;\log{\hat{Y}}_{i,j}$$

where ${\hat{Y}}_{i,j}$ is the predicted probability that graph $G_{i}$ belongs to class j, and the $Y_{i,j}$ denotes the ground truth label.

### 3.2 Brain Structure Inference

As discussed in Section 2.1, the Granger causality algorithm, a prevalent tool for inferring causal relationships among brain regions, has two primary limitations. First, it can only determine the existence of causality and cannot quantify the strength of causal effects. Second, when assessing the presence of causality between two brain regions, the algorithm overlooks the potential influence of other brain regions on the pair being analysed. To address the limitations of the traditional GC algorithm, we propose a novel Generative Adversarial Network (GAN) model, named Brain GAN, to estimate causal effects among all brain regions while considering all elements simultaneously.

The detailed network structure of Brain GAN is illustrated in Figure 2, which incorporates multiple generative modules to evaluate the causal effects among brain regions based on the input time series data $X\in R^{t\times v}$. Here, t represents the number of time points, while v corresponds to the number of brain regions. In the field of causal inference, there is a consensus that causes produce effects. According to this principle, the causal effect of other brain regions on the h‐th brain region can be defined as follows:

(3) $${\hat{X}}_{:,h}=\tanh\left(\sum_{h=1}^{v}G_{h,i}X_{:,h}\right)$$

where $G_{h,i}$ denotes the causal effects of the h‐th brain region on the i‐th brain region.

**FIGURE 2:**
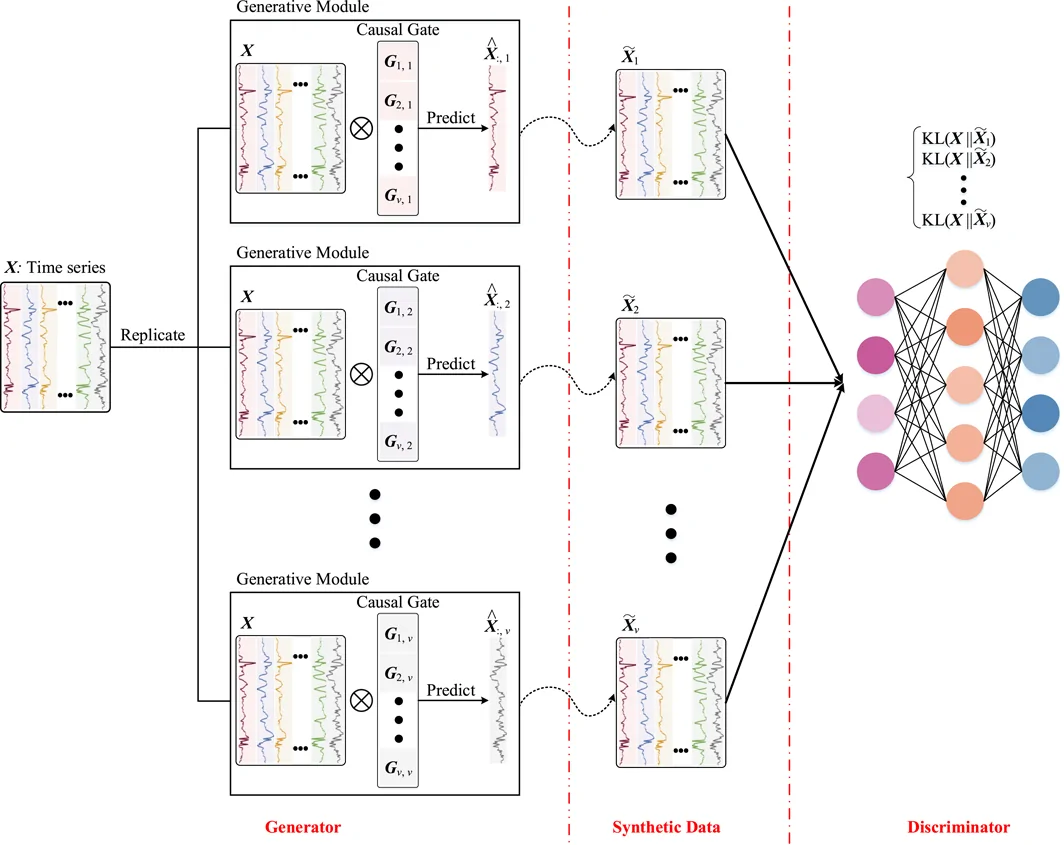
The detailed structure of Brain GAN. First, the time series data X is input into the generative module to identify the causes associated with the corresponding brain region. Next, each column of the time‐series data X is replaced with the estimated signal, denoted as ${\tilde{X}}_{i}$. Finally, a discriminator network is utilised to distinguish between real samples and synthetic samples.

To evaluate the causal effects among all brain regions, we developed a novel generative network composed of v generative modules. The i‐th generative module $f_{i}$ consists of a causal gate $G_{:,i}\in R^{v}$, which is used to preserve the causal effects of all other brain regions on the i‐th brain region. The generator network comprises v generative modules, allowing it to infer the causal effects among all brain regions simultaneously.

For the i‐th generative module, the predicted BOLD signal for the i‐th brain region, denoted as ${\hat{X}}_{:,i}$, can be derived. A synthetic sample, denoted as ${\tilde{X}}_{i}$, can be created by replacing the actual BOLD signal of a specific brain region, represented as $X_{:,i}$, with the generated BOLD signal for the corresponding brain region, ${\hat{X}}_{:,i}$. Given that the generator network consists of v generative modules, each subject can produce v synthetic samples through one‐to‐one replacement, specifically ${\tilde{X}}_{1},{\tilde{X}}_{2},\text{…},{\tilde{X}}_{v}$.

To enhance the realism of the predicted BOLD signals $\hat{X}$ relative to actual signal values X, we integrated a shared discriminative network to ensure that, even with extensive training, the model cannot confidently differentiate between synthetic and real samples. The loss function for Brain GAN can be derived as follows:

(4) $$\begin{array}{ccc} G_{\mathrm{loss}} & = & \|\hat{X}-X{\|}_{2}^{2}+\frac{1}{v}\sum_{i=1}^{v}C_{:,i}\\ D_{\mathrm{loss}} & = & \frac{1}{v}\sum_{s=1}^{v}D_{KL}\left(X\|{\tilde{X}}_{i}\right) \end{array}$$

where $G_{\mathrm{loss}}$ is the generator loss, the introduction of the sum of causal effects $C_{:,i}$ aims to achieve optimal fitting performance while maintaining low complexity. $D_{\mathrm{loss}}$ denotes the discriminator loss, which is used to minimise the differences in data distribution between the synthetic sample ${\tilde{X}}_{i}$ and the real sample X by reducing the Kullback–Leibler divergence.

### 3.3 Hierarchical Representation Learning

Graph Neural Networks (GNNs) have attracted more and more attention due to their superior ability to represent graph‐structured data. Extensive research indicates that extracting multi‐level representations can enhance the performance of downstream tasks. Thus, we have developed a novel GNN, termed the Hierarchical Representation Graph Neural Network (HGNN), to extract hierarchical representations from brain networks. The detailed structure of HGNN is illustrated in Figure 1, which comprises three graph convolution layers and three graph pooling layers.

The graph convolution operation for the k‐th layer is defined as follows:

(5) $$N^{k+1}=\sigma \left({\tilde{D}}^{-\frac{1}{2}}{\tilde{G}}^{k}{\tilde{D}}^{-\frac{1}{2}}N^{k}W^{k}\right)$$

where ${\tilde{G}}^{k}$ represents the sub‐graph of the brain network G after k−1 layers, $N^{k}$ denotes the node feature matrix subsequent to k−1 layers. Both ${\tilde{G}}^{k}$ and $N^{k}$ serve as inputs to the k‐th graph convolution layer. The function σ(⋅) is a non‐linear activation function. Additionally, $\tilde{D}$ is the diagonal degree matrix of ${\tilde{G}}^{k}$, and $W^{k}$ is a trainable weight matrix.

The objective of the graph pooling operation is to preserve the essential structure of the graph as much as possible while reducing its scale. In this paper, we utilise the widely adopted graph pooling operation known as the TopK pooling strategy to execute downstream operations [26]. The graph pooling operation for the k‐th layer can be expressed as follows:

(6) $$\begin{array}{ccc} index & = & \text{TopIndex}\;\left(N^{k},p\right)\\ G^{k+1} & = & G_{\mathrm{index,index}}^{k} \end{array}$$

where the function TopIndex(⋅) returns the indices of the top p values according to node importance. $G^{k+1}$ is the updated brain network following to the k‐th graph pooling layer.

Finally, the representations of different subgraphs are aggregated to form a final representation for identifying patients with dementia. The aggregated graph representation $G_{r}$ is subsequently input into a Multi‐Layer Perceptron (MLP) layer with a softmax classifier for graph classification. The cross‐entropy loss function is employed for training the HGNN.

## 4 Experimental Setup

In this section, we first present the demographic information of the dataset used in this study in Section 4.1, followed by the training details for Brain GAN and HRGCN in Section 4.2.

### 4.1 Data and Preprocessing

In this study, we utilised resting‐state fMRI data from 214 subjects obtained from the publicly available Alzheimer's Disease Neuroimaging Initiative (ADNI) database.^1^ The subjects included normal controls (NC), early mild cognitive impairment (eMCI) and late mild cognitive impairment (LMCI). Demographic information is presented in Table 1. All human subjects involved in this research were sourced from ADNI, and each participant provided written informed consent after receiving a comprehensive written and verbal explanation of the study. The research was approved by the Research Ethics Board of ADNI.^2^

**TABLE 1:** Demographic information of the used dataset.

Group	NC	eMCI	LMCI
Male/Female	28/39	32/45	36/34
Age(mean ± STD)	74.1±6.2	71.2±6.9	71.4±6.8

For fMRI data preprocessing, we followed standard procedures as outlined below. First, we discarded the initial five volumes from each subject to eliminate noise signals, retaining the remaining 135 volumes for subsequent analysis. All functional images were registered to the first image and transformed into Montreal Neurological Institute (MNI) space, with a resampled voxel size of 3× 3× 3 mm^3^. Subsequently, we utilised the CONN Toolbox^3^ to perform the following steps: direct segmentation, normalisation, linear detrending, and functional smoothing, among others. Finally, we extracted the time series for each brain region from the preprocessed images based on the AAL atlas.

### 4.2 Model Training

For the Brain GAN, each generative module $f_{i}$ is configured as a one‐hidden‐layer neural network with 100 tanh units. The causal gate $C_{:,i}$ is randomly initialised, except for the self‐loop terms $C_{i,i}$, which are set to 0. The shared discriminator network is designed as a two‐hidden‐layer neural network with 100 hidden ReLU units. The Adam optimiser, with a learning rate of 0.001, is employed to optimise the model. The generator network is trained for 3000 epochs, while the discriminator network is trained for 1000 epochs.

For the HGNN, the Adam optimiser is utilised to optimise the network. The learning rate is set to 0.001, and the model is trained for 1000 epochs. The pooling value p in Equation (6) is explored within the range of {0.1,0.2,…,0.9}. The MLP consists of two fully connected layers, with the number of units in each layer set to 64 and 32, respectively, followed by a softmax classifier.

Notably, to determine the hyperparameters (hidden units and epochs), we reserved 20% of the training set as a validation set. For the generator, we performed a grid search over the number of hidden units: 32, 64, 100 and 128. The recognition performance with 100 units was comparable to that with 128 units during the generator epochs. Therefore, 100 units were selected as the optimal balance between model capacity and generalisation, while also reducing the number of training parameters. For the training epochs, we adopted an early stopping strategy with a patience of 50 epochs, monitoring the validation loss. The maximum epoch caps (3000 for the generator, 1000 for the discriminator, and 1000 for HGNN) were set as upper bounds. However, the actual converged epochs were approximately 800, 560, and 600, respectively. Additionally, $L_{2}$ weight decay was applied to the hidden layers of the generator to further mitigate overfitting.

## 5 Results

In this section, we first provide a detailed description of the comparison methods outlined in Section 5.1. Then, we present the experimental results in Section 5.2. Finally, an ablation study is conducted in Section 5.3 to demonstrate the effectiveness of the proposed method.

### 5.1 Comparison Methods

In this paper, we propose two neural networks, Brain GAN and HGNN, to enhance the traditional framework for recognising neurological disorders. Brain GAN is employed to estimate causal effects among brain regions, with the objective of improving conventional brain network modelling algorithms. Conversely, HGNN is an advanced GNN designed to obtain multilevel representations of brain networks, offering a significant improvement over traditional classifiers.

To validate the efficacy of the proposed brain network modelling algorithm, Brain GAN, we conducted a comparative analysis with six widely used brain network modelling algorithms: (1) Pearson correlation (PC) [11, 12, 15], (2) sparse brain network (SBN) [27], (3) low‐rank brain network (LRBN) [12], (4) sparse low‐rank brain network (SLRBN) [12], (5) Granger causality (GC) [28], and (6) multivariate Granger causality (mGC) [11, 12, 15]. The first four methods define the interaction relationships among brain regions from a correlation perspective, while the latter two methods, along with our proposed Brain GAN, evaluate these relationships from a causality perspective. In this study, the weights of brain network edges were used as features as inputs into the classifier to obtain prediction outcomes.

To test the effectiveness of the graph classifier HGNN in recognising dementia, we conducted a comparative analysis with two commonly used classifiers: random forest (RF) [29] and support vector machine (SVM) [30]. The RF parameters were configured with 500 trees and a maximum depth of 3. For the SVM, we set the parameters to 200 iterations, a linear kernel and a regularisation parameter of 1.0. The pooling value p for HGNN was established at 40; the rationale for this choice can be found in Section 5.3.

To quantify the performance of various algorithms in recognising dementia cases, four widely used metrics were employed: accuracy (ACC), sensitivity (SEN), specificity (SPE), and the F1 score. To enhance the reliability of the classification results, a 10‐fold cross‐validation strategy was utilised.

To evaluate the effectiveness of the proposed model, the dataset was randomly partitioned at the subject level into three independent subsets to prevent data leakage between training and testing. A stratified random sampling strategy was employed to maintain the original proportions of the NC, eMCI, and LMCI groups across all subsets. The data were split into 60% for training, 20% for validation, and 20% for testing. The training set was used for model parameter optimisation, the validation set for hyperparameter tuning, and the testing set was strictly reserved for final classification performance evaluation. To reduce variability caused by random partitioning, all experiments were repeated using five distinct random seeds (i.e., five independent random subsampling iterations), and the average performance along with the corresponding standard deviations are reported.

### 5.2 Experimental Results

Table 2 presents the recognition performances of all methods across three classifiers, with the best scores highlighted in bold. Our proposed method consistently achieves optimal classification accuracy in most instances, demonstrating its efficacy in simultaneously estimating causal effects among brain regions. In addition, two points are noteworthy. First, among the four correlation‐based methods, LRBN yields the best results in most cases, potentially due to the incorporation of prior information [12]. In contrast, SLRBN frequently has the lowest performance, likely because it introduces numerous constraints that render the estimated brain network overly sparse, thereby excluding many effective features. Second, causality‐based methods generally outperform correlation‐based methods, achieving an accuracy of over 80% in many instances. This suggests that causality‐based methods are more effective in estimating interaction relationships among brain regions, which may be attributed to their ability to eliminate spurious connections [17, 31, 32].

**TABLE 2:** Recognition performance of all methods across three different classifiers.

Classifier	Model	NC vs. eMCI				NC vs. LMCI				eMCI vs. LMCI			
		ACC	SEN	SPE	F1	ACC	SEN	SPE	F1	ACC	SEN	SPE	F1
RF	PC	70.03	67.45	71.88	69.27	77.35	72.11	80.58	74.68	75.78	77.46	**70.18**	76.52
	SBN	72.15	62.57	80.14	67.53	74.36	72.34	76.85	75.07	65.35	72.32	56.84	67.35
	LRBN	79.68	76.21	84.26	78.34	78.25	74.37	80.33	75.94	**78.41**	83.85	70.65	**79.87**
	SLRBN	69.51	60.64	76.85	64.35	78.42	77.03	78.72	77.53	63.95	75.12	53.44	68.48
	GC	78.56	70.07	**88.53**	76.28	81.64	76.95	84.86	80.25	64.44	67.86	62.37	66.45
	mGC	79.07	68.25	87.62	75.42	81.16	**80.22**	83.48	81.36	61.48	72.36	52.08	68.35
	Ours	**81.64**	**76.38**	85.06	**78.96**	**83.15**	77.28	**88.09**	**81.25**	76.65	**85.47**	67.02	79.32
SVM	PC	70.42	67.61	72.57	69.91	75.31	71.29	78.41	73.69	67.34	65.21	71.26	67.75
	SBN	75.11	70.24	79.35	72.48	77.42	74.73	80.15	76.47	70.12	81.93	57.26	74.22
	LRBN	82.73	79.24	85.87	81.03	84.08	79.25	**88.69**	82.91	81.75	85.82	77.27	83.14
	SLRBN	73.75	70.28	76.74	71.36	76.75	74.72	78.66	75.87	65.46	81.97	47.26	71.23
	GC	80.64	79.25	81.95	79.26	80.35	79.26	81.54	79.83	69.44	74.12	64.33	71.76
	mGC	82.05	74.72	**88.46**	79.43	82.55	**85.15**	80.12	82.73	81.05	78.06	**84.31**	81.15
	Ours	**83.42**	**79.28**	87.14	**81.68**	**85.47**	82.15	**88.69**	**84.72**	**83.01**	**87.15**	78.62	**84.38**
HGNN	PC	75.83	73.21	78.04	73.75	76.71	82.12	71.57	77.53	71.52	66.36	77.24	70.98
	SBN	78.54	71.73	84.57	75.62	78.13	82.15	74.36	78.64	75.53	84.52	65.83	78.34
	LRBN	79.91	76.25	83.23	77.94	81.02	76.27	85.83	79.74	79.63	84.54	74.32	81.31
	SLRBN	73.74	71.75	75.43	71.74	74.53	68.74	80.13	72.54	71.54	72.83	70.14	72.73
	GC	82.73	80.64	84.53	81.24	81.03	76.24	85.84	79.73	77.54	80.53	74.34	78.93
	mGC	82.04	77.73	**85.73**	80.04	83.24	79.13	**87.14**	82.13	78.24	83.14	72.93	80.03
	Ours	**84.03**	**82.14**	**85.73**	**82.74**	**84.63**	**82.19**	**87.14**	**83.94**	**83.64**	**85.73**	**81.43**	**84.63**

*Note:* The bold values represent the best performance (maximum value) for each evaluation metric (ACC, SEN, SPE, F1) within each column.

Comparing the three classifiers, it is evident that the proposed HGNN model outperforms traditional classifiers across various scenarios. This superiority can be attributed to two primary factors. First, HGNN is a flexible GNN with feature extraction capabilities. Second, the proposed method effectively extracts hierarchical features from brain regions, thereby further enhancing classification performance. The classification results presented in Table 2 demonstrate the effectiveness of our proposed framework.

To further validate the effectiveness of the proposed method, we compared it with several causality‐based approaches using a simulated dataset. The synthetic data, initially generated with dynamic causal models (DCMs) as detailed by Smith et al. [33], are widely used for benchmarking the accuracy of effective connectivity estimation. These data exhibit nonstationary connectivity strengths among brain regions that vary over time, driven by additional random processes.^4^ For evaluation, we selected three representative simulation scenarios to test the proposed method: Sim1, which involves nonlinear dynamics; Sim2, which incorporates shared external inputs; and Sim3, which introduces a global mean confound across all nodes.

We adopted four standard metrics to quantitatively evaluate the performance of each method in causal structure recovery: Precision, Recall, F1‐score, and Structural Hamming Distance (SHD). Precision quantifies the proportion of correctly identified causal edges among all detected edges, reflecting the method's ability to avoid false positive connections. Recall measures the proportion of true causal edges that are successfully recovered, indicating the method's sensitivity in capturing existing effective connectivity. The F1‐score, as the harmonic mean of precision and recall, provides a balanced overall assessment of the model's accuracy in causal inference. In contrast, SHD calculates the total number of structural discrepancies—including missing edges, spurious edges, and reversed edges—between the estimated network and the ground‐truth causal graph. For Precision, Recall, and F1‐score, higher values indicate superior causal structure recovery performance, while a lower SHD value signifies a smaller deviation from the true network and thus indicates better estimation accuracy.

As shown in Table 3, the proposed method consistently outperforms the baseline approaches (GC, mGC) across all three simulated scenarios. The traditional Granger causality (GC) method achieves moderate recall in Sim1 and Sim3; however, its precision and F1‐score are lower than those of the proposed method. Additionally, its Structural Hamming Distance (SHD) values are consistently higher, indicating a large number of false positive connections and structural errors. The modified Granger causality (mGC) method, when integrated with the HGNN classifier, exhibits extremely poor recall and F1‐score across all scenarios, accompanied by notably elevated SHD values, demonstrating its inability to effectively capture true causal relationships under complex dynamic conditions. In contrast, our method attains the highest precision, recall, and F1‐score, along with the lowest SHD across Sim1, Sim2, and Sim3. This superiority is particularly evident in challenging scenarios: in Sim2, which involves shared external inputs, our method improves the F1‐score by 19.1% and reduces SHD by 29.2% compared to GC; in Sim3, characterised by a global mean confound, it achieves an F1‐score of 0.74%–18.9% higher than GC—and an SHD of 2.00, the lowest among all methods. These results indicate that the proposed method effectively mitigates the limitations of traditional causality estimation approaches, robustly recovers accurate causal brain networks under diverse confounding conditions, and demonstrates excellent generalisation and reliability for effective connectivity analysis.

**TABLE 3:** Recognition performance of three methods on the simulated dataset.

Classifier	Model	Sim1				Sim2				Sim3			
		Precision	Recall	F1	SHD	Precision	Recall	F1	SHD	Precision	Recall	F1	SHD
HGNN	GC	0.56	0.72	0.63	2.01	0.45	0.51	0.48	3.35	0.43	0.79	0.55	2.18
	mGC	0.60	0.25	0.32	3.78	0.47	0.29	0.35	3.89	0.65	0.31	0.38	3.66
	Ours	**0.68**	**0.76**	**0.70**	**1.72**	**0.62**	**0.77**	**0.67**	**2.37**	**0.72**	**0.78**	**0.74**	**2.00**

### 5.3 Ablation Study

It is important to highlight that the graph pooling operation in the HGNN model includes a hyperparameter, namely the pooling value p. The value of p has a significant impact on the performance of downstream tasks. Setting p too high can result in the retention of excessive redundant and irrelevant information, whereas setting it too low may cause the loss of critical information. To address this issue, we conducted ablation experiments on the hyperparameter p, with the objective of identifying the optimal value within the range of [10–80].

Figure 3 illustrates how recognition performance varies as the value of p is adjusted across the three tasks. As illustrated in Figure 3, the recognition performance of the HGNN model exhibits a distinct trend across the three tasks. As the parameter p increases from 10 to 40, the model's recognition performance improves continuously, reaching its peak when p=40. However, with further increments in p, the model's recognition performance gradually declines. This observation indicates that p=40 is an optimal balance value. Comparing the three curves, we can find that when the value of p ranges from 40 to 80, the decline in recognition performance between the tasks (NC vs. LMCI) is relatively minor. This may be attributed to the substantial differences between the normal population and patients with late MCI. Although excessively high p values may introduce irrelevant information, the significant differences between the two groups mitigate the negative impact on classification performance.

**FIGURE 3:**
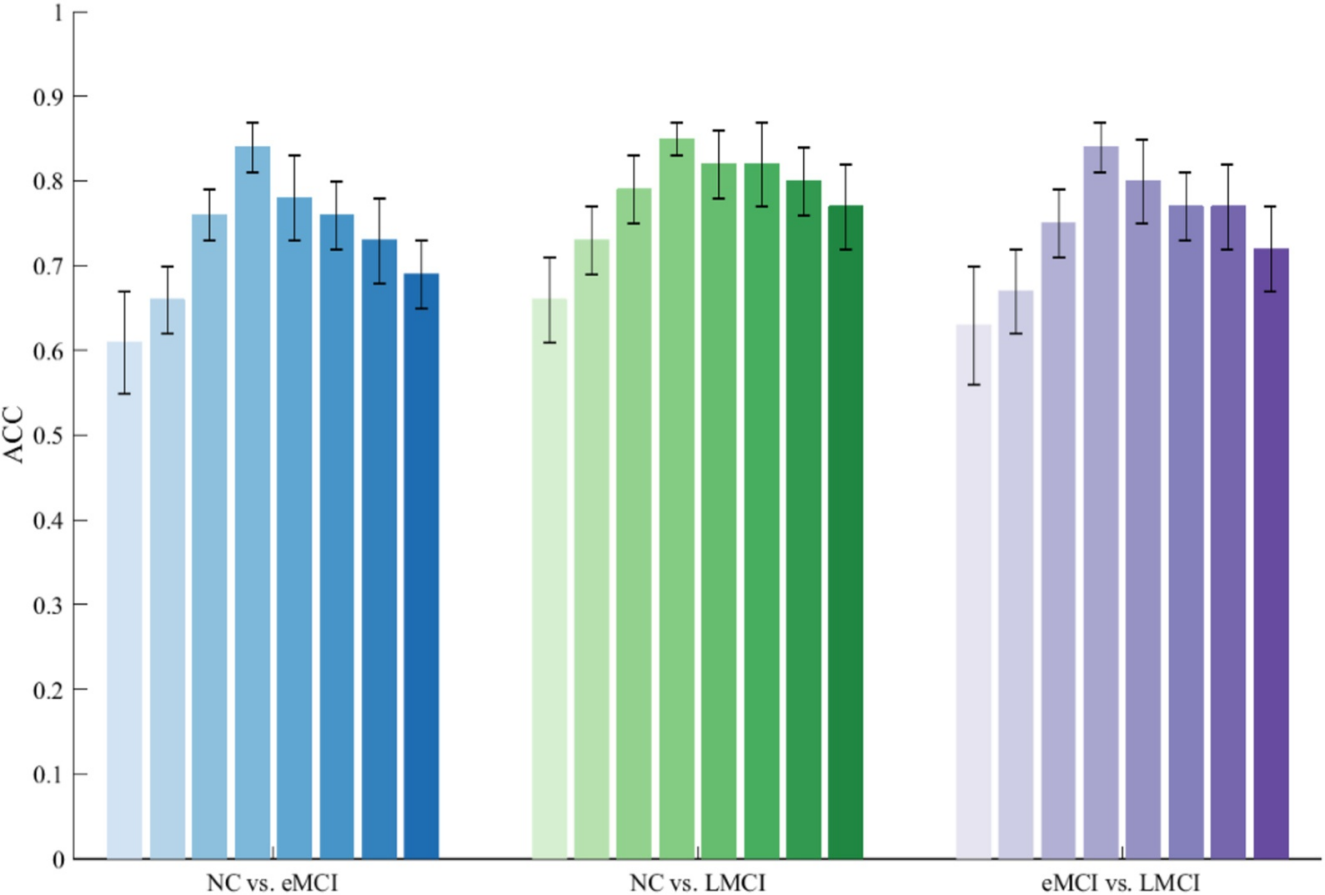
The impact of various p values on the classification performance of HGNN across three classification tasks. For each group, the leftmost bar represents the classification result at p=10. As one progresses towards the rightmost column, the value of p gradually increases, culminating in a value of 80.

In conclusion, the ablation experiments demonstrate the significant impact of varying p values on the recognition performance of the HGNN model. When p is set to 40, the HGNN model can retain sufficient information for the downstream task.

In summary, from a biological perspective, our causal brain network model discards unrealistic assumptions such as linearity and isolated pairwise analysis, aligning instead with the true physiological mechanisms of directed information transmission between brain regions. It accurately reveals the direction of neural information flow and abnormal causal interaction patterns, providing a more biologically interpretable foundation for investigating the pathological mechanisms underlying neurological disorders. From a clinical perspective, the proposed method improves the accuracy of brain disease identification and can uncover new potential biomarkers. It is expected to serve as an effective auxiliary tool for the early and differential diagnosis of neurological disorders. The identified abnormal causal connections also help locate key pathological circuits, offering valuable references for targeted interventions and precise treatments in clinical practice.

## 6 Discussion

### 6.1 Most Discriminative Patterns

Figure 4 illustrates the most discriminative connectivity patterns across the three tasks. The Circos graph shows the top 10 weighted connections derived from the SVM classifier. There are three key points to note regarding the Circos graph. First, the colour of each arc is randomly assigned solely for visualisation. Second, the width of each arc corresponds to the number of connections associated with the respective brain region. Finally, the movement of the small ball within the Circos graph reflects the causal relationship between two brain regions, indicating the flow from cause to effect.

**FIGURE 4:**
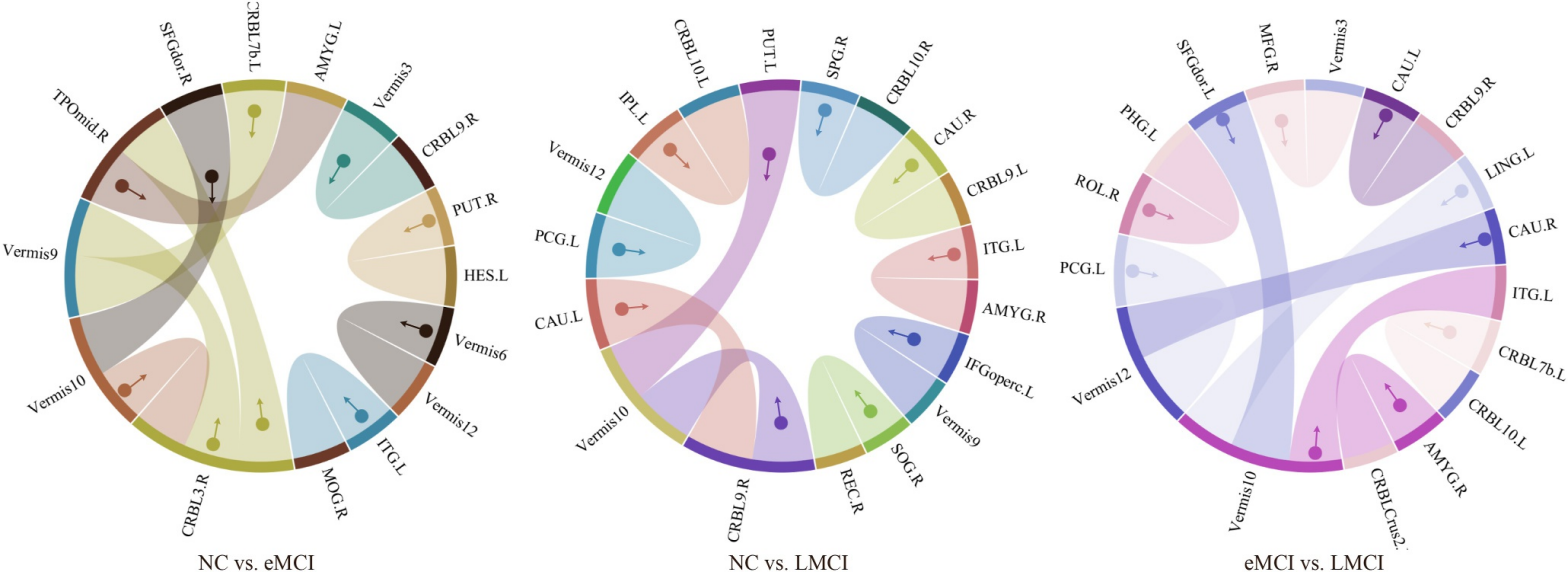
The discriminative connectivity patterns for different tasks (from left to right: NC vs. eMCI, NC vs. LMCI and eMCI vs. LMCI).

From Figure 4, we can observe that many of the identified brain regions in the Circos graph correspond with findings reported in previous studies. For instance, the inferior temporal gyrus Jie et al. [34], Song et al. [35], the lenticular nucleus Jie et al. [34], Qiu et al. [36], Song et al. [35], and the amygdala Chen et al. [37], Khazaee et al. [38] are frequently reported to be strongly associated with dementia. Additionally, our results suggest that Vermis10 may also be significantly correlated with the onset of dementia, as abnormalities in connections associated with this brain region were observed across all three tasks.

The three sets of differentially altered brain connections identified in this study, derived from effective connectivity analyses, reveal the spatiotemporal reorganisation of brain network function across the continuum from normal cognition (NC) to early and late mild cognitive impairment (eMCI and LMCI) in Alzheimer's disease. In the comparison between NC and eMCI, significant changes primarily involve cerebellar subregions, the limbic system (e.g., amygdala), the temporal lobe, and subcortical nuclei, indicating early disruption of cerebellar‐cognitive and limbic circuits that may mark the initial shift in cognitive function. In the comparison between NC and LMCI, abnormal connections become widely distributed across multiple large‐scale networks, including the default mode network and the salience network, consistent with global cognitive decline and indicative of progression towards Alzheimer's disease. In the comparison between eMCI and LMCI, progressive alterations predominate in cerebellar‐subcortical and limbic‐temporal circuits, which may serve as dynamic markers for monitoring MCI progression.

These findings enhance the physiological understanding of Alzheimer's disease (AD) from a network perspective. First, they confirm early functional abnormalities in the cerebellum that worsen as the disease progresses, underscoring the importance of including the cerebellum in AD pathophysiological models—an aspect traditionally underemphasised in prior studies. Second, the directed information flow patterns revealed by effective connectivity illustrate a spatiotemporal trajectory of network dysfunction, beginning in limbic regions and extending to widespread cortical areas.

## 7 Conclusion

The current algorithms employ a pairwise matching approach to estimate the causal relationships between brain regions. However, this methodology often neglects the potential influence of other brain regions on the assessment. To address this limitation, we introduce an innovative network model that considers all relevant brain regions when evaluating causal effects among them. Compared with existing methods, the proposed approach demonstrates superior performance across various tasks with different classifiers, highlighting its efficacy. Furthermore, this study validates frequently reported biomarkers and identifies novel biomarkers, such as Vermis10, offering new perspectives and insights into related research fields.

## Author Contributions

**Junhong Ren:** data curation, formal analysis, methodology, software, validation, visualisation, writing – original draft, writing – review and editing. **Wensheng Zhang:** conceptualisation, funding acquisition, investigation, methodology, project administration, supervision, writing – review and editing.

## Funding

This research was funded by the National Key Research and Development Program of China (Grant 2023YFC3604600).

## Ethics Statement

Since the data used in this article are from a public dataset, ethical statements are not applicable.

## Conflicts of Interest

The authors declare no conflicts of interest.

## Data Availability

Data underlying the results of this study are available in the Alzheimers Disease Neuroimaging Initiative (ADNI) (URL: http://adni.loni.ucla.edu).

## Endnotes

^1^

http://adni.loni.ucla.edu.

^2^

http://adni.loni.ucla.edu/study‐design/ongoing‐investigations/.

^3^

https://web.conn‐toolbox.org/.

^4^

https://www.fmrib.ox.ac.uk/datasets/netsim/index.html.
